# Incidence of C5 nerve root palsy after cervical surgery: A meta-analysis for last decade

**DOI:** 10.1097/MD.0000000000008560

**Published:** 2017-11-10

**Authors:** Tao Wang, Hui Wang, Sen Liu, Wen-Yuan Ding

**Affiliations:** a Department of Orthopedics, Wuxi NO. 9 People's Hospital Affiliated to Soochow University, Wuxi; b Department of Spinal Surgery, The Third Hospital of Hebei Medical University, Shijiazhuang, China.

**Keywords:** C5 nerve root palsy, incidence, meta-analysis

## Abstract

**Purpose:**

We aim to perform a meta-analysis on incidence of C5 nerve root palsy (C5 palsy) for patients after cervical surgery.

**Methods:**

An extensive search of the literature was performed in PubMed/MEDLINE, Embase, the Cochrane library, CNKI, and WANFANG databases on incidence of C5 palsy from January 2007 to January 2017. Prevalence of C5 palsy related to different surgery methods was calculated and data analysis was conducted with STATA 12.0.

**Results:**

A total of 61 studies containing 721 patients with C5 palsy in total 11,481 patients (6.3%) were included in our study. The incidences after anterior cervical discectomy and fusion (ACDF), anterior cervical corpectomy and fusion (ACCF), anterior corpectomy combined with discectomy (ACCDF), laminoplasty (LP) and laminectomy and fusion (LF) were 5.5%, 7.5%, 6%, 4.4%, and 12.2%, respectively. Compared with anterior approaches (5%), female patients (4%) and patients with cervical spondylotic myelopathy (CSM) (4.8%), posterior approaches (6.2%), male patients (5.7%) and patients with ossification of posterior longitudinal ligament (OPLL) (8.1%) have a higher prevalence. In ACDF and LP, patients with OPLL (5.5%, 8.1%, respectively) have a higher incidence than those in patients with CSM (4.7%, 3.1%, respectively); however, in LF, patients with CSM and OPLL have similar incidence of C5 palsy (13% vs 13.1%). In most cases, C5 palsy was unilateral (74.5%).

**Conclusions:**

Based on our meta-analysis, posterior approaches, male patients and patients with OPLL have a higher incidence of C5 palsy. In ACDF and LP, patients with OPLL have a higher incidence of C5 palsy, but in LF, patients with CSM and OPLL have similar result.

## 1 Introduction

C5 nerve root palsy (C5 palsy) is a common complication after cervical surgery including anterior and posterior approaches: anterior cervical discectomy and fusion (ACDF), anterior cervical corpectomy and fusion (ACCF), anterior corpectomy combined with discectomy (ACCDF), laminoplasty (LP), and laminectomy and fusion (LF), which was reported first by Scoville.^[1]^ Shou et al^[2]^ reported that the incidence of C5 palsy was 5.3% (95% CI 4.6%–6.0%). Sakaura et al^[3]^ showed that prevalence of C5 palsy varied from 0% to 30%. Patients with C5 palsy had paresis of the deltoid muscle and/or the biceps brachii muscle after surgery without any deterioration of myelopathic symptoms.^[4,5]^

Previous studies that reported the posterior decompression were easier to cause C5 palsy compared with the anterior decompression.^[6,7,8]^ But the reason remained controversial. Few hypotheses reported that the spinal cord or nerve root may lead to C5 palsy. Some believed that nerve root traction caused by the cord shifting resulted in C5 nerve root lesion after posterior decompression surgery, which is called “tethering phenomenon.”^[9–11]^ Another hypothesis was that spinal cord lesion caused by acute decompression and expansion of the spinal cord lead to C5 plasy.^[12–14]^ Hence, it is necessary to review studies related to C5 palsy for concluding the incidence of C5 palsy after all kinds of surgeries and it can give some valuable comments for spinal surgeons.

Previous reviews^[2,8,15]^ on the prevalence and risk factors of C5 palsy have few included studies or some included studies in the 1990s and 2000s, which was far from now. So, we performed a meta-analysis on incidence of C5 palsy for the last decade.

## 2 Materials and methods

### 2.1 Ethics statement

There is no need to seek informed consent from patients, since this is a meta-analysis based on the published data, without any potential harm to the patients; this is approved by Ethics Committee of The Third Hospital of HeBei Medical University.

### 2.2 Search strategy

An extensive search of literature was performed in PubMed, Embase, the Cochrane library, CNKI, and WANFANG databases. The following key words were used for search: “C5 never root palsy,” “cervical,” “anterior cervical discectomy and fusion,” “anterior cervical corpectomy and fusion,” “corpectomy combined with discectomy,” “laminoplasty,” “laminectomy and fusion,” “cervical spondylotic myelopathy,” and “ossification of posterior longitudinal ligament” from January 2007 to January 2017, with various combinations of the operators “AND” and “OR.” Language was restricted to Chinese and English.

### 2.3 Inclusion criteria

Studies were included if they met the following criteria: randomized or nonrandomized controlled study; age greater than or equal to 18 years old; studies on C5 palsy after cervical surgery.

### 2.4 Exclusion criteria

Studies were excluded if they met the following criteria: had repeated data; did not report outcomes of interest; in vitro human cadaveric biomechanical studies; earlier trial, reviews, and case-reports.

### 2.5 Selection of studies

All subjects, abstracts, and the full text of articles were reviewed independently by 2 reviewers. According to the inclusion criteria, we selected the eligible trials. If disagreement occurred, we consulted the third reviewer to resolve the disagreement.

### 2.6 Data extraction and management

Two reviewers extracted independently data. The data extracted including the following categories: study ID, study design, study location, total patients, follow-up, mean age, gender, incidence of C5 palsy after anterior or posterior approaches including ACDF, ACCF, ACCDF, LP, and LF, sex of patients with C5 palsy.

### 2.7 Statistical analysis

We used STATA 12.0 to analyze data (Stata Corporation, College Station, TX). Both were reported with 95% confidence intervals (CI) and a *P* value of.05 was applied as the level of statistical significance. We used I^2^ tests to assess statistical heterogeneity, which was from 0% to 100% in meta-analysis assessments. When I^2^ >50% among the included studies, we chose random effects model; if not, we chose fixed-effects model.^[17,18]^

### 2.8 Test for risk of publication bias

We used a visual inspection of the funnel plot to assess publication bias. If there is publication bias, the funnel plot should be asymmetric, if not, the funnel plot is symmetric. We also performed the Egger and Begg tests to measure the funnel plot asymmetry by using a significance level of *P*<.05. Additionally, we applied trim and fill computation to estimate the effect of publication bias.

## 3 Results

### 3.1 Search results

**Figure 1:**
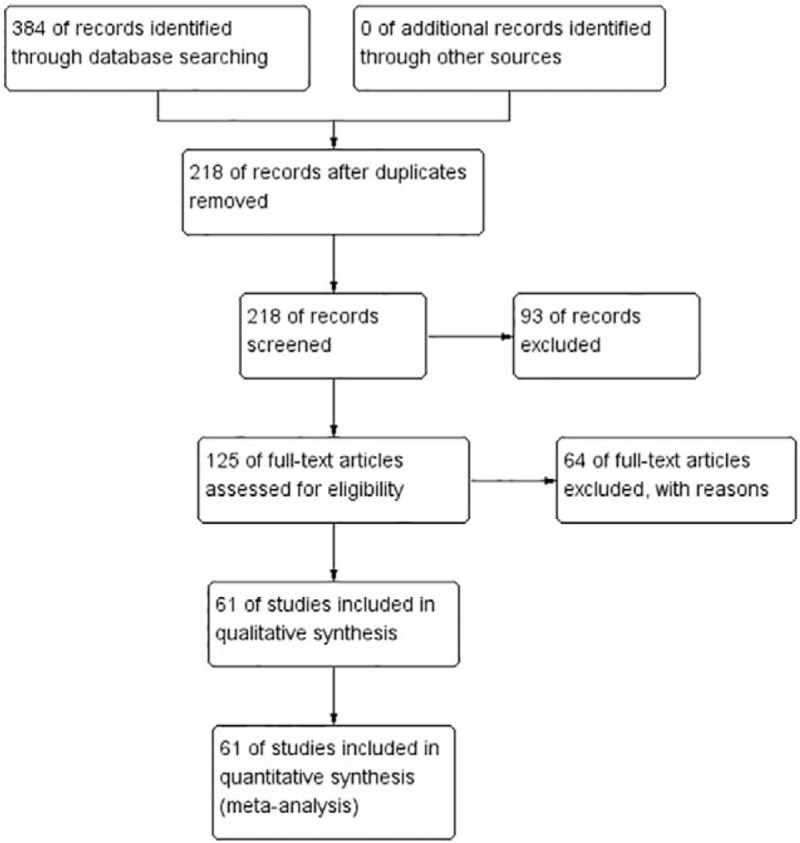
Flow diagram of study selection.

We had searched 321 English studies in MEDLINE, EMBASE, 63 Chinese studies in WANFANG and CNKI. Of these, 136 English articles and 30 Chinese after duplicates removed, 80 English articles and 13 Chinese articles were excluded due to unrelated studies. Fifty-four English articles and 10 Chinese article were excluded due to eligibility criteria. As a result, a total of 61 studies were identified for this meta-analysis. The literature search procedure was shown in Fig. 1.

### 3.2 Baseline characteristics and quality assessment

A total of 61 studies including 721 patients with C5 palsy in total 11,481 patients (6.3%) were included in our study. Baseline characteristics of included articles are shown in Table 1.

**Table 1:**
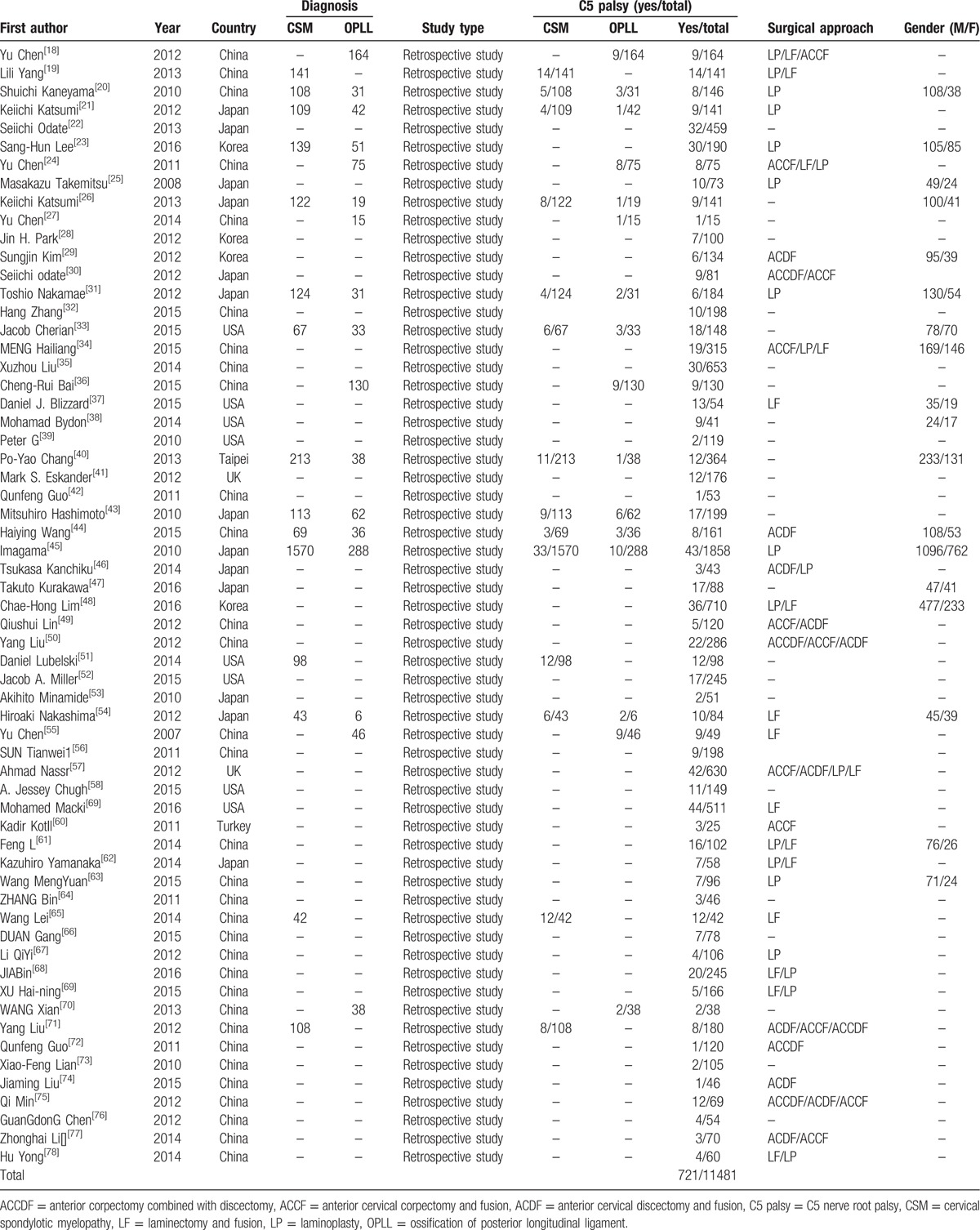
Characteristics of included studies.

All included studies were retrospective studies, we used the Newcastle Ottawa Quality Assessment Scale to assess the quality of each study. This scale for nonrandomized case controlled studies and cohort studies were used to allocate a maximum of 9 points for the quality of selection, comparability, exposure, and outcomes for study participants. Of these studies, 53 studies scored 8 points and 8 studies scored 7 points. Hence, the quality of each study was relatively high (Table 2 ).

**Table 2:**
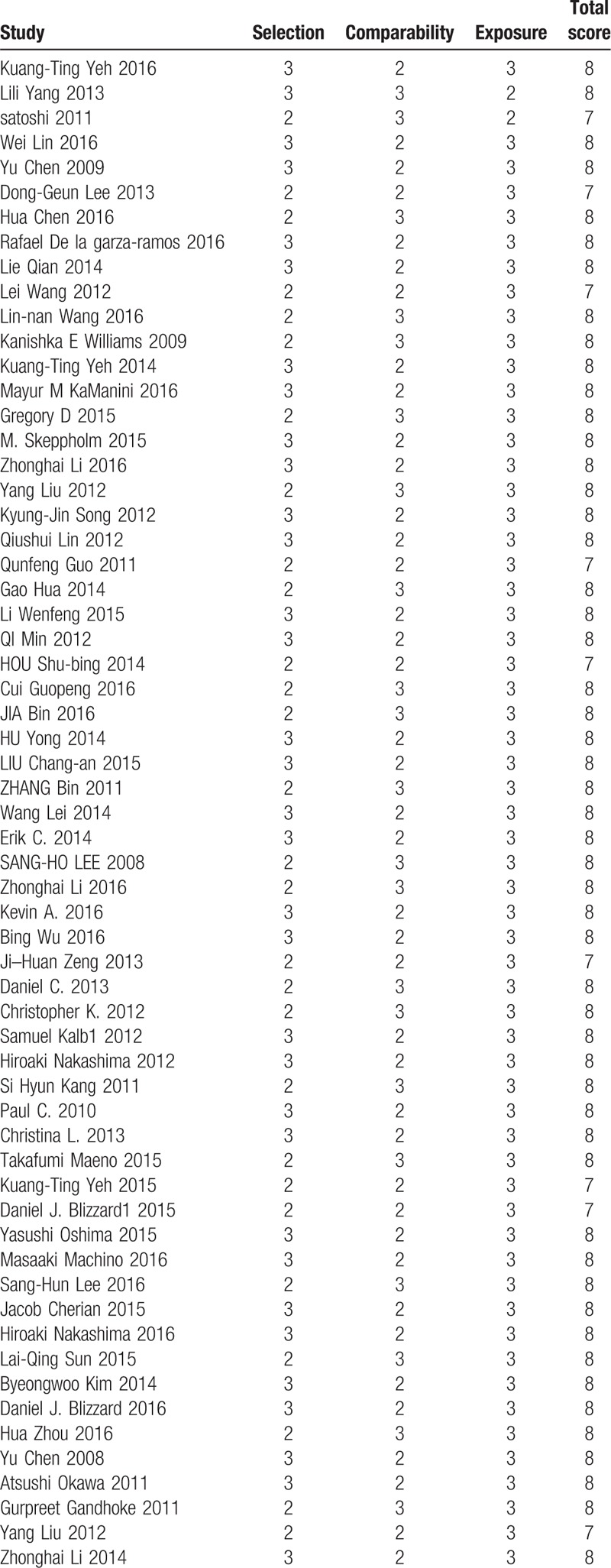
The quality assessment according to the Newcastle Ottawa Quality Assessment Scale (NOQAS) of each study.

**Table 2 (Continued):**
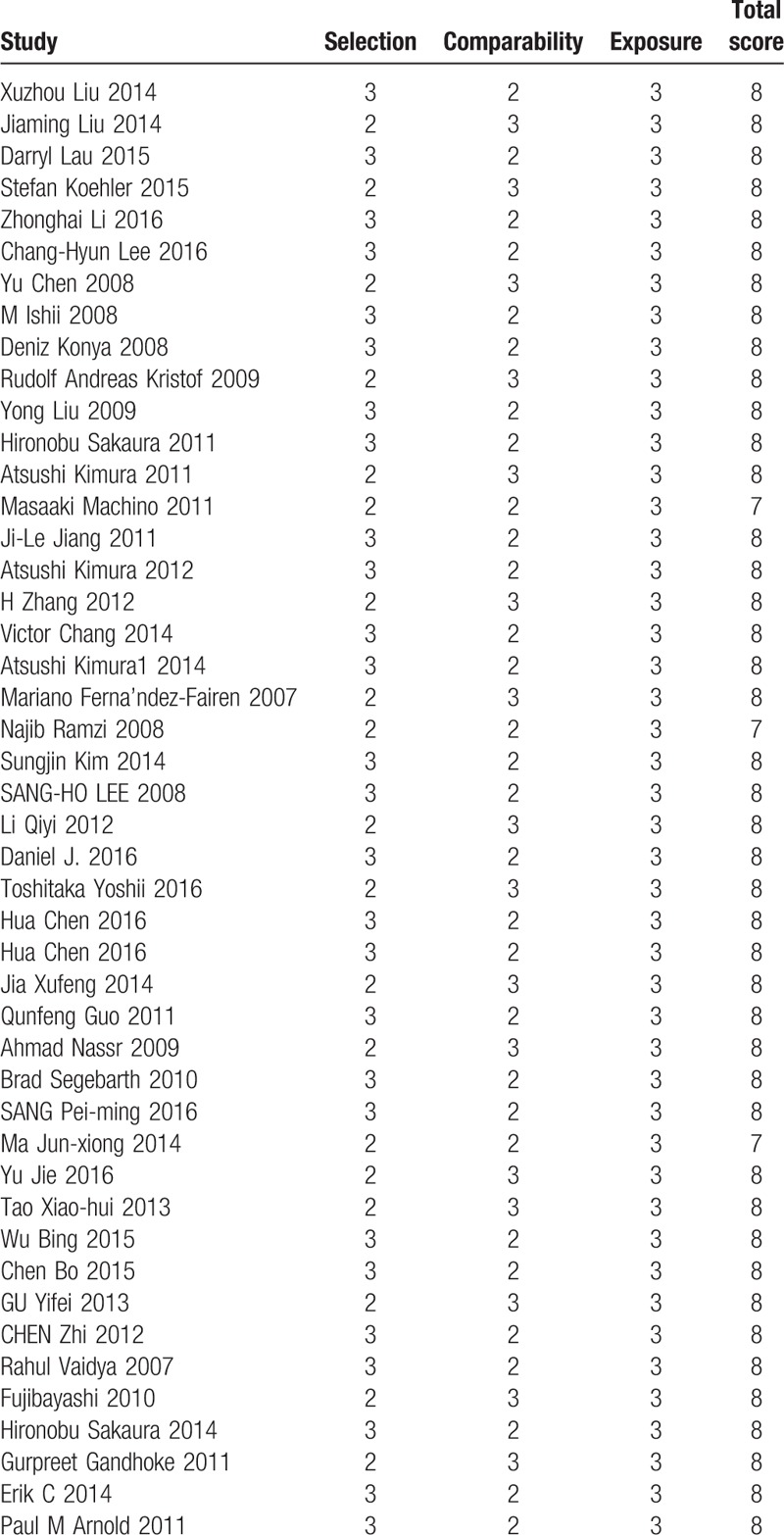
The quality assessment according to the Newcastle Ottawa Quality Assessment Scale (NOQAS) of each study.

### 3.3 Overall prevalence of C5 palsy

**Figure 2:**
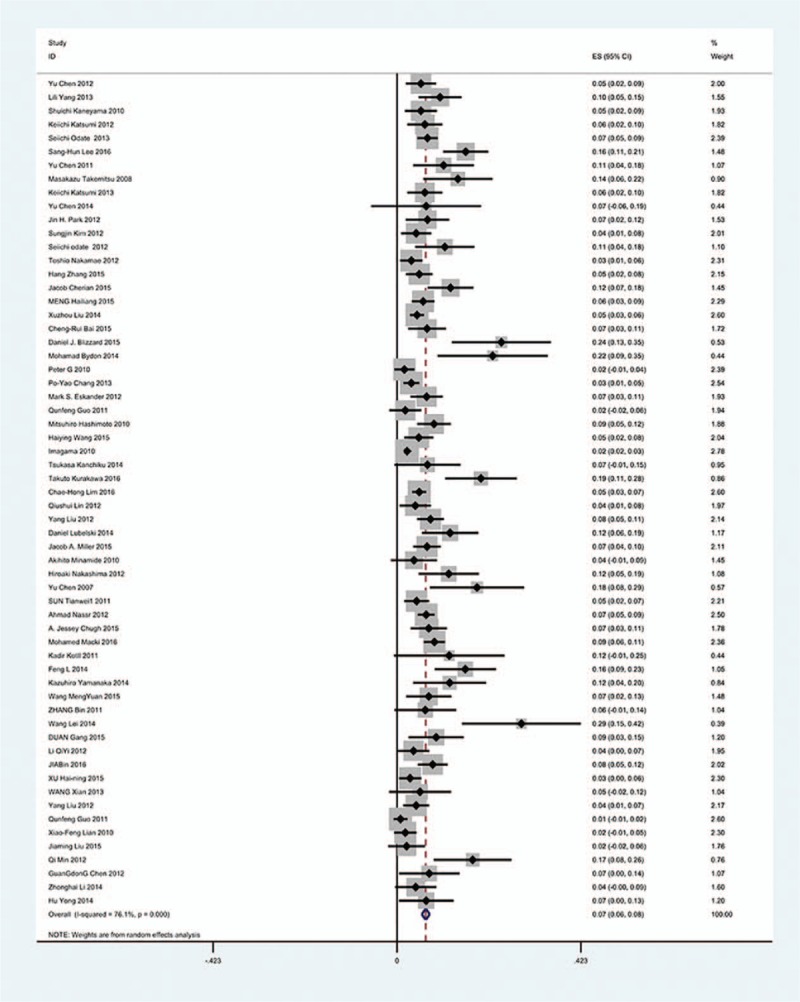
Forest plot showing incidence of C5 after cervical surgery. CI = confidence interval, df = degrees of freedom, M–H = Mantel–Haenszel.

Sixty-one studies^[18–78]^ containing total 11,481 patients 721 patients with C5 palsy after cervical surgery were included for meta-analysis. Figure 2 shows that the incidence of C5 palsy was 6.3% (95% CI 5.7%–7.9%), with substantial heterogeneity of incidence observed. The incidence of C5 palsy among the studies varied between 1% and 29%.

### 3.4 Surgical approaches-related C5 palsy

**Figure 3:**
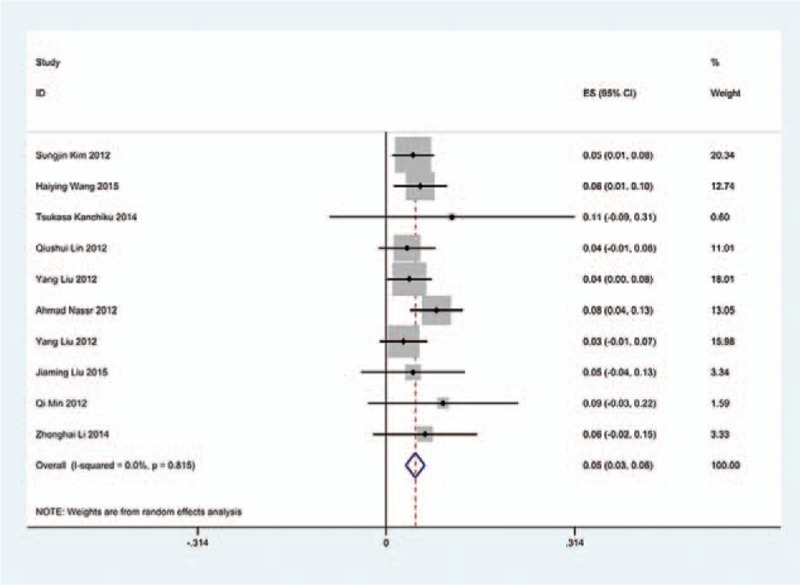
Forest plot showing incidence of C5 after ACDF. ACDF = anterior cervical discectomy and fusion, CI = confidence interval, df = degrees of freedom, M–H = Mantel–Haenszel.

**Figure 4:**
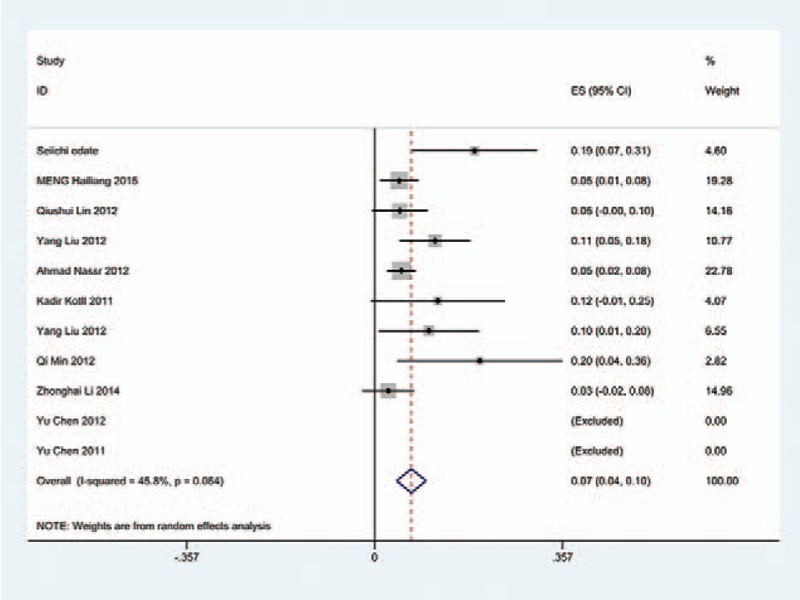
Forest plot showing incidence of C5 after ACCF. ACCF = anterior cervical corpectomy and fusion, CI = confidence interval, df = degrees of freedom, M–H = Mantel–Haenszel.

**Figure 5:**
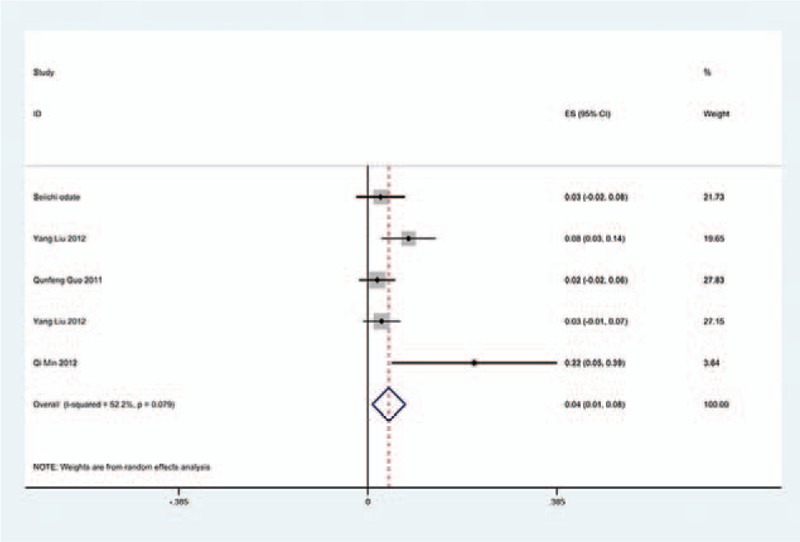
Forest plot showing incidence of C5 after ACCDF. ACCDF = anterior corpectomy combined with discectomy, CI = confidence interval, df = degrees of freedom, M–H = Mantel–Haenszel.

**Figure 6:**
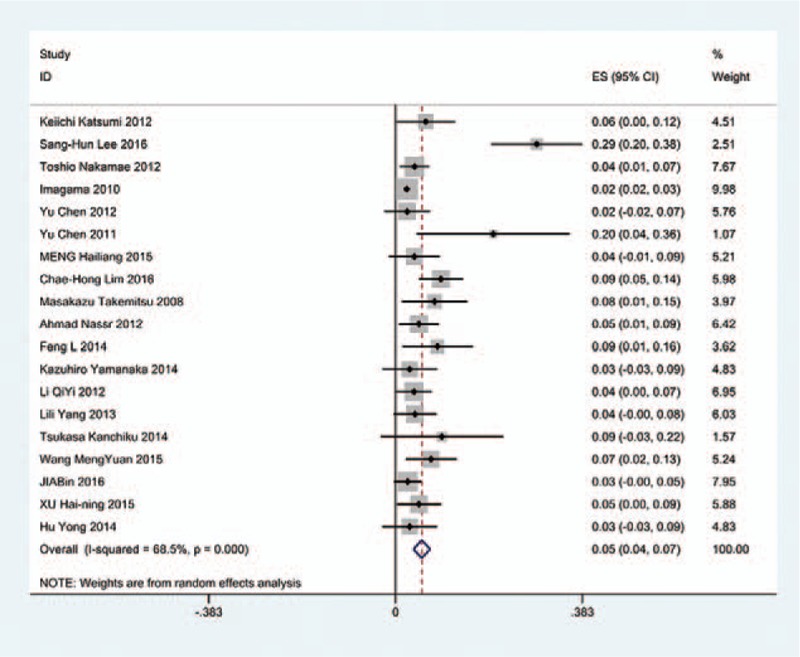
Forest plot showing incidence of C5 after LP. CI = confidence interval, df = degrees of freedom, LP = laminoplasty, M–H = Mantel–Haenszel.

**Figure 7:**
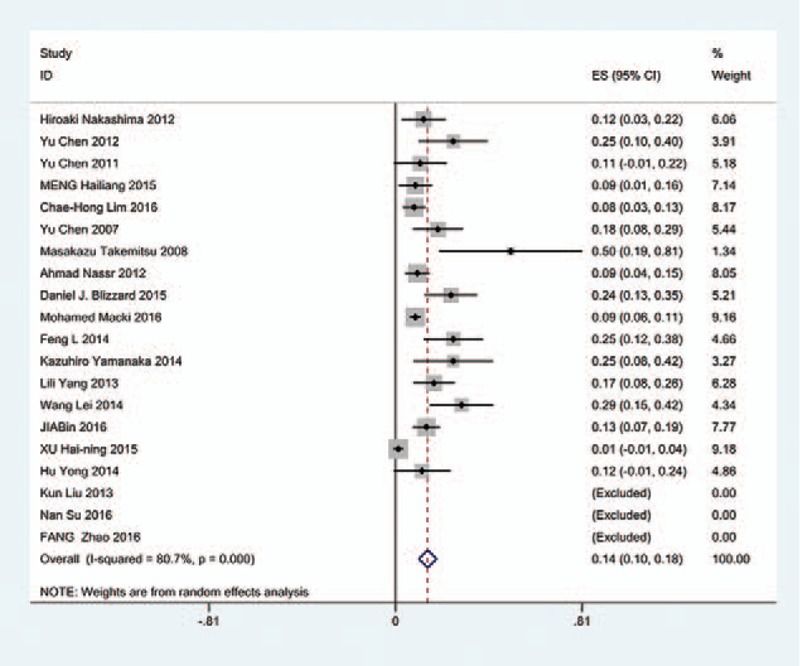
Forest plot showing incidence of C5 after LF. CI = confidence interval, df = degrees of freedom, LF = laminectomy and fusion, M–H = Mantel–Haenszel.

**Figure 8:**
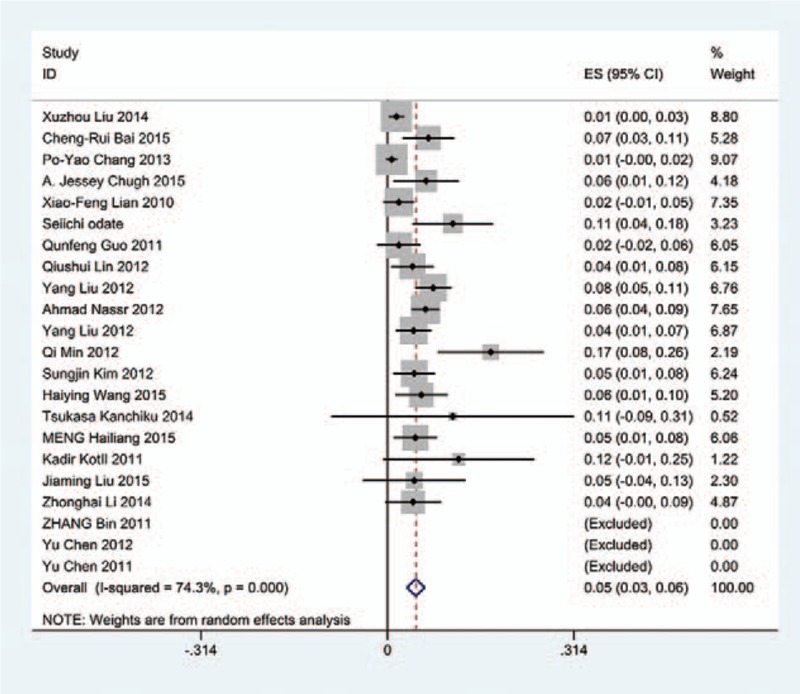
Forest plot showing incidence of C5 after anterior surgery. CI = confidence interval, df = degrees of freedom, M–H = Mantel–Haenszel.

**Figure 9:**
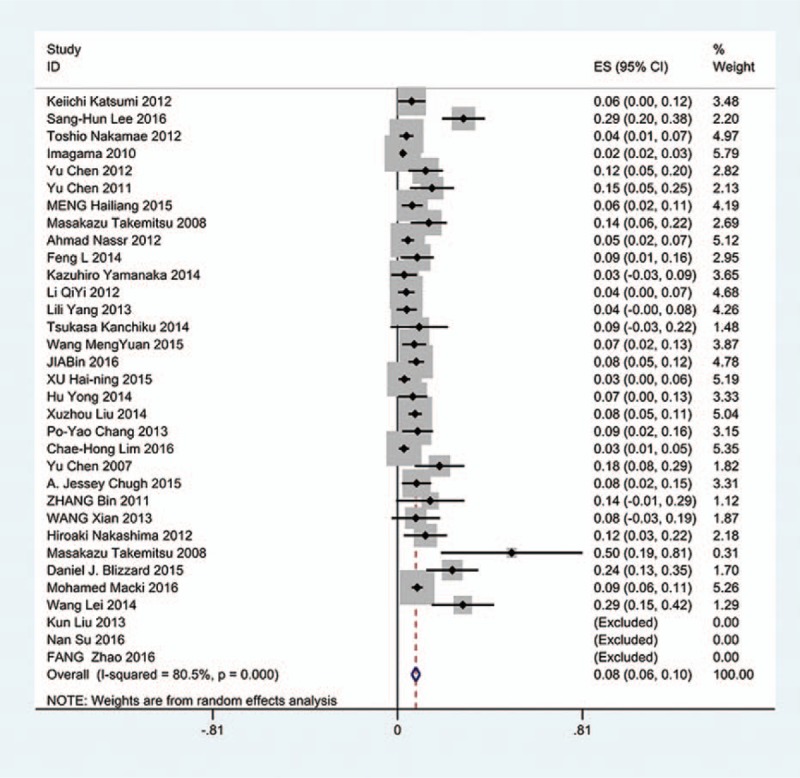
Forest plot showing incidence of C5 after posterior surgery. CI = confidence interval, df = degrees of freedom, M–H = Mantel–Haenszel.

The results revealed that anterior approaches (5%) have a lower incidence of C5 palsy than these in posterior approaches (6.2%). As for all kinds of surgical methods, patients with ACDF (5.5%) have the lowest incidence of C5 palsy and LF (13%) have the highest incidence of C5 palsy. The incidences of C5 palsy for ACCF, ACCDF, and LP were 7.5%, 6%, 4.4%, respectively (Figs. 3–9).

### 3.5 Diseases type-related C5 palsy

**Figure 10:**
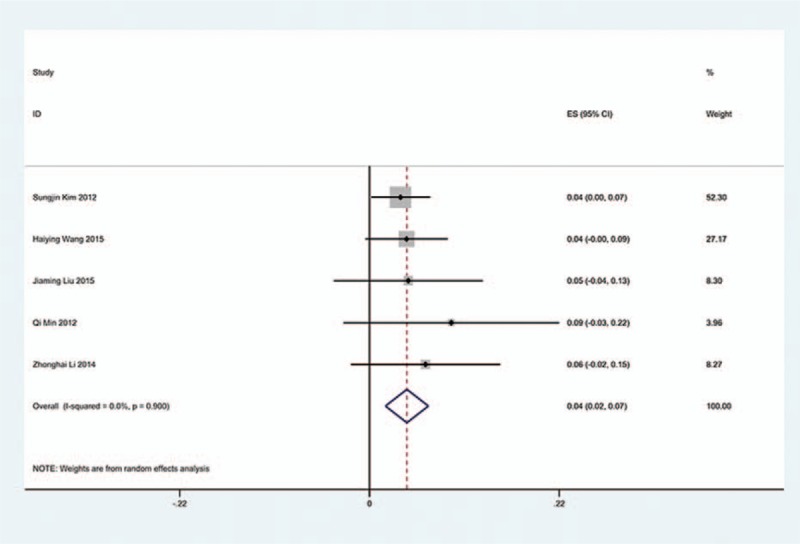
Forest plot showing incidence of C5 for patients with CSM after ACDF. ACDF = anterior cervical discectomy and fusion, CI = confidence interval, CSM = cervical spondylotic myelopathy, df = degrees of freedom, M–H = Mantel–Haenszel.

**Figure 11:**
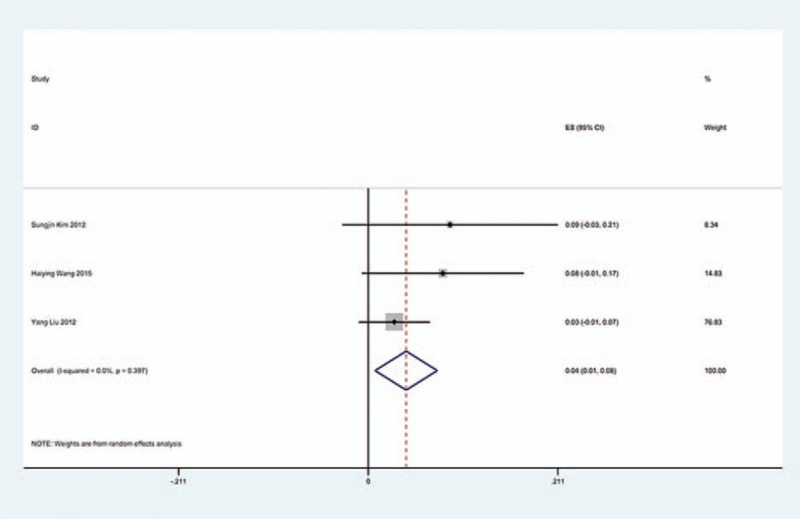
Forest plot showing incidence of C5 for patients with OPLL after ACDF. ACDF = anterior cervical discectomy and fusion, CI = confidence interval, CSM = cervical spondylotic myelopathy, df = degrees of freedom, M–H = Mantel–Haenszel, OPLL = ossification of posterior longitudinal ligament.

**Figure 12:**
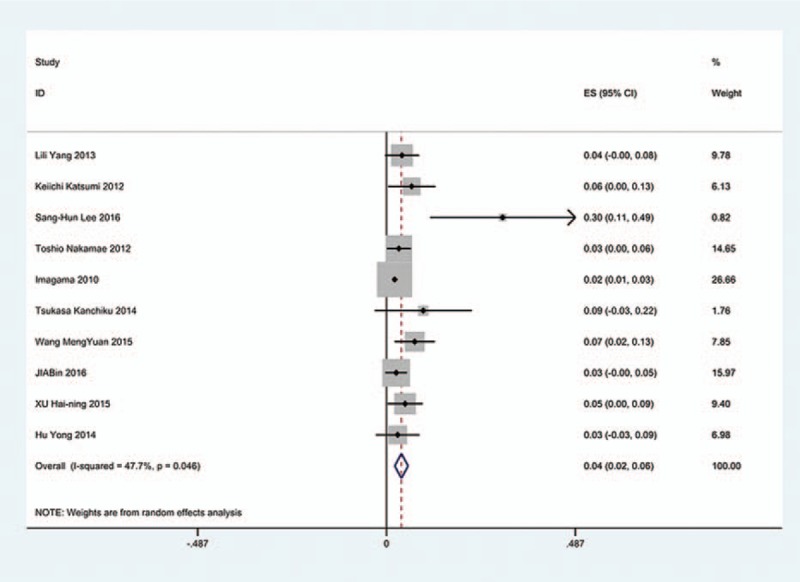
Forest plot showing incidence of C5 for patients with CSM after LP. CI = confidence interval, df = degrees of freedom, LP = laminoplasty, M–H = Mantel–Haenszel.

**Figure 13:**
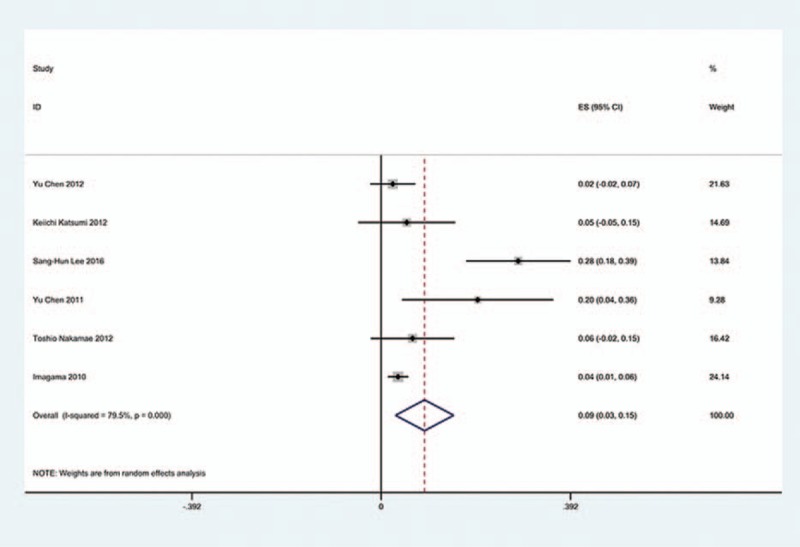
Forest plot showing incidence of C5 for patients with OPLL after LP. CI = confidence interval, df = degrees of freedom, LP = laminoplasty, M–H = Mantel–Haenszel, OPLL = ossification of posterior longitudinal ligament.

**Figure 14:**
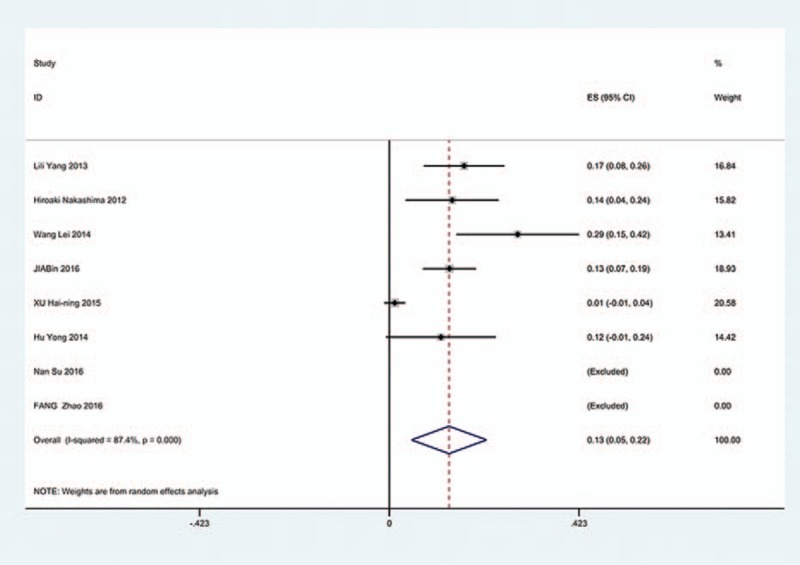
Forest plot showing incidence of C5 for patients with CSM after LF. CI = confidence interval, CSM = cervical spondylotic myelopathy, df = degrees of freedom, LF = laminectomy and fusion, M–H = Mantel–Haenszel.

**Figure 15:**
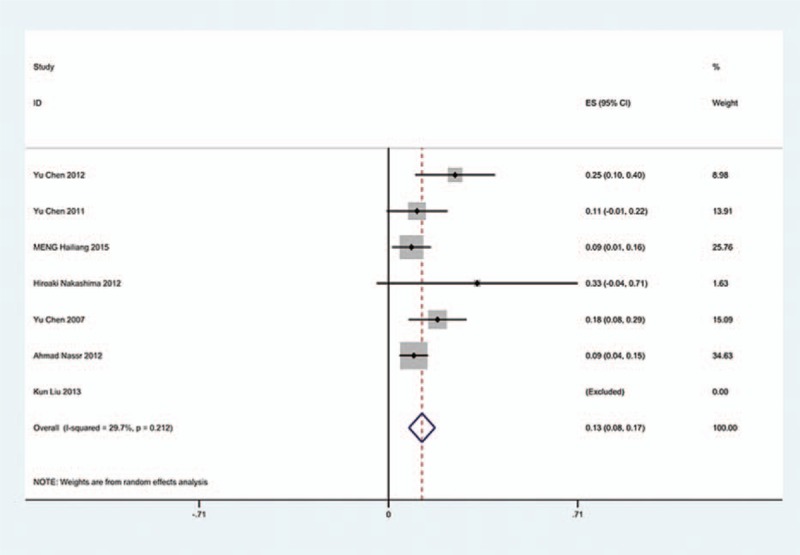
Forest plot showing incidence of C5 for patients with OPLL after LF. CI = confidence interval, df = degrees of freedom, M-H = Mantel-Haenszel.

**Figure 16:**
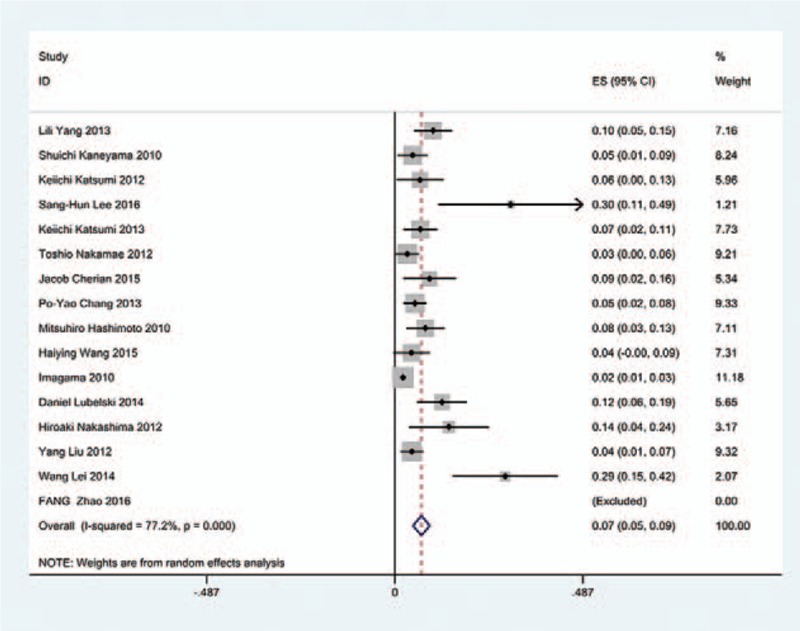
Forest plot showing incidence of C5 for patients with CSM after cervical surgery. CI = confidence interval, CSM = cervical spondylotic myelopathy, df = degrees of freedom, M–H = Mantel–Haenszel.

**Figure 17:**
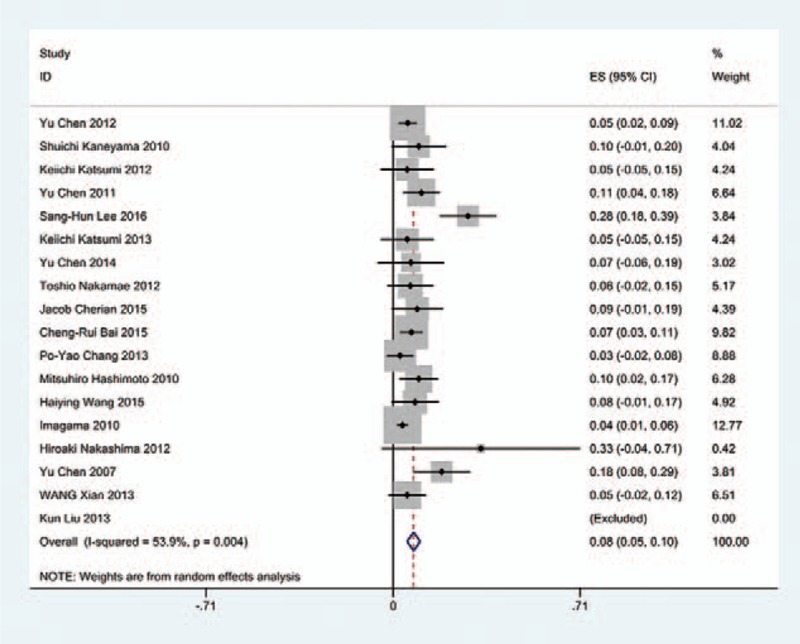
Forest plot showing incidence of C5 for patients with OPLL after cervical surgery. CI = confidence interval, df = degrees of freedom, M–H = Mantel–Haenszel, OPLL = ossification of posterior longitudinal ligament.

We only computed the incidence of C5 palsy for CSM and OPLL, because other cervical diseases lack enough data. The results presented that patients with OPLL (8.1%) have a higher incidence of C5 palsy than patients with CSM (4.8%). We found that, in ACDF and LP, patients with OPLL (5.5%, 8.1%, respectively) have a higher incidence than those in patients with CSM (4.7%, 3.1%, respectively); however, in LF, patients with CSM and with OPLL have similar incidence of C5 palsy (13% vs 13.1%) (Figs. 10–17).

### 3.6 Gender-related C5 palsy

**Figure 18:**
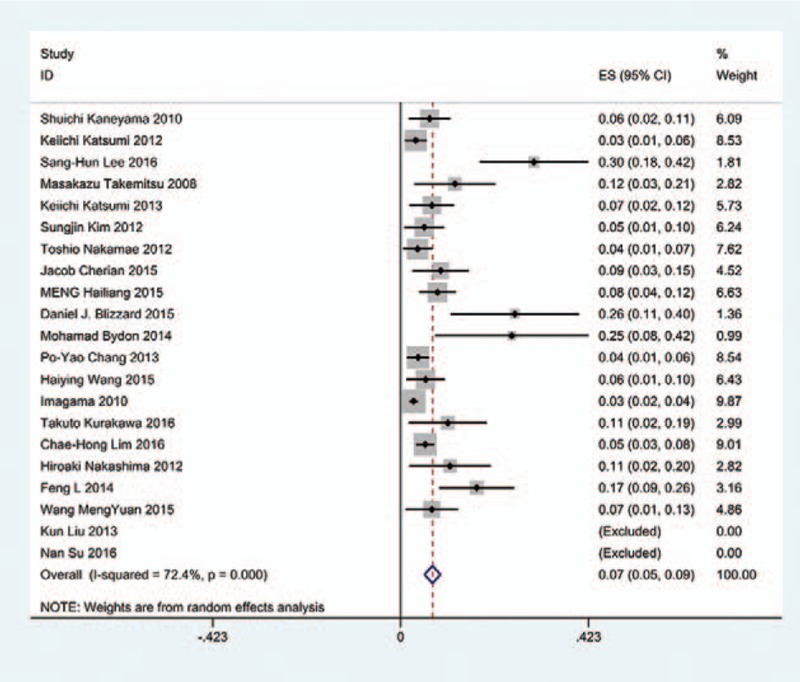
Forest plot showing incidence of C5 for male patients after cervical surgery. CI = confidence interval, df = degrees of freedom, M–H = Mantel–Haenszel.

**Figure 19:**
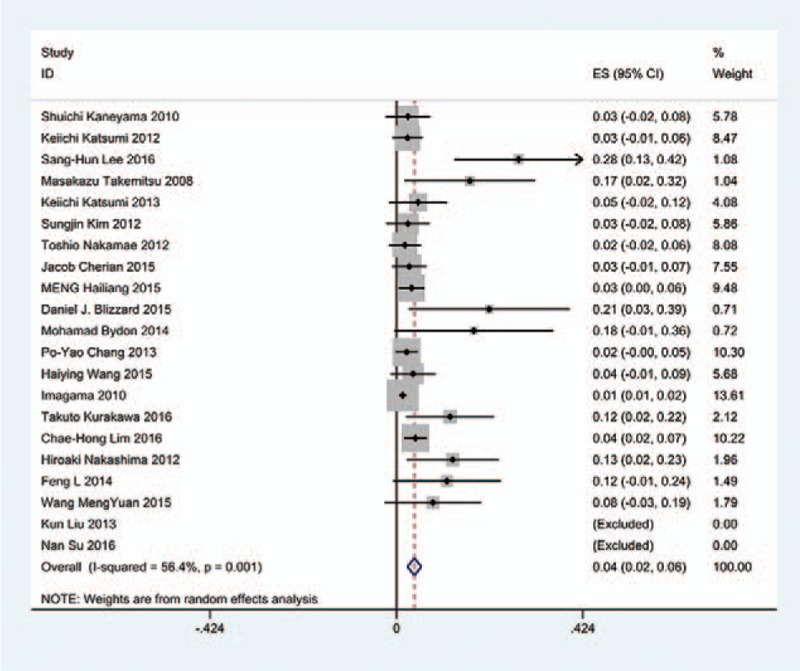
Forest plot showing incidence of C5 for female patients after cervical surgery. CI = confidence interval, df = degrees of freedom, M–H = Mantel–Haenszel.

Figures 18 and 19 reveal that the prevalence of C5 palsy in males has higher incidence than that in females (5.9% vs. 4.1%).

### 3.7 Sides-related C5 palsy

**Figure 20:**
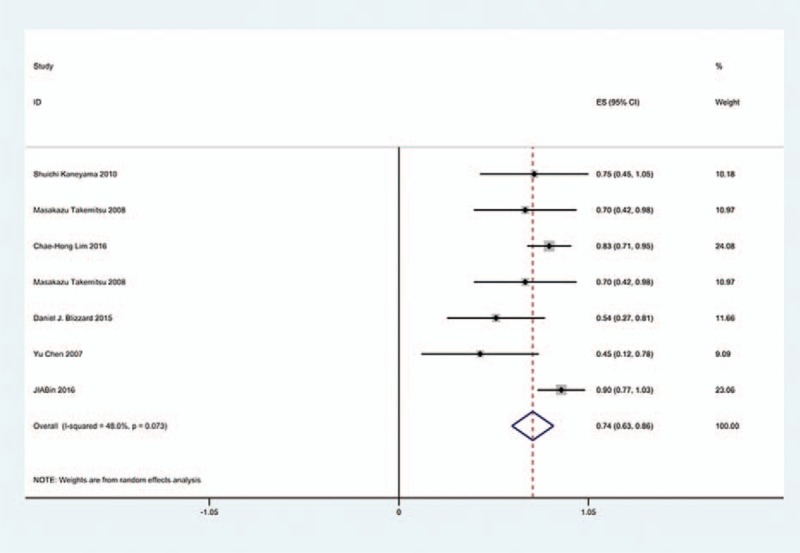
Forest plot showing incidence of unilateral C5 after cervical surgery. CI = confidence interval, df = degrees of freedom, M–H = Mantel–Haenszel.

**Figure 21:**
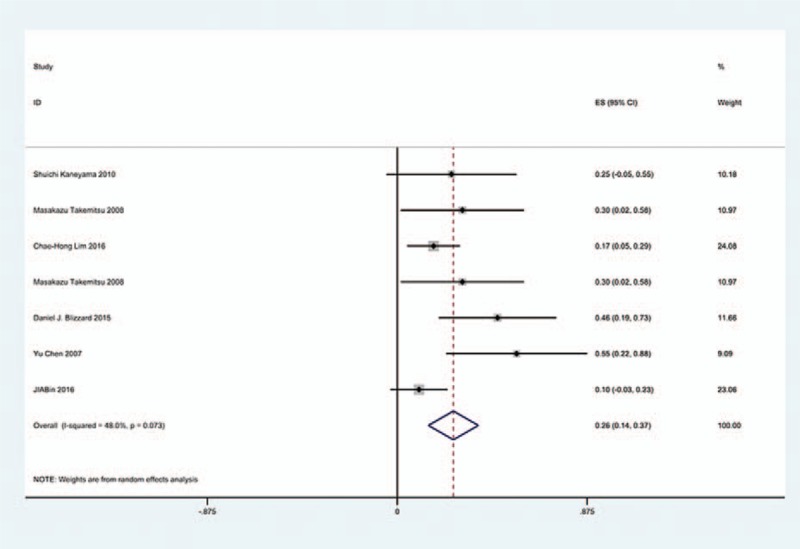
Forest plot showing incidence of bilateral C5 after cervical surgery. CI = confidence interval, df = degrees of freedom, M–H = Mantel–Haenszel.

Figures 20 and 21 suggest that most cases of C5 palsy were unilaterally involved.

### 3.8 Publication bias

**Figure 22:**
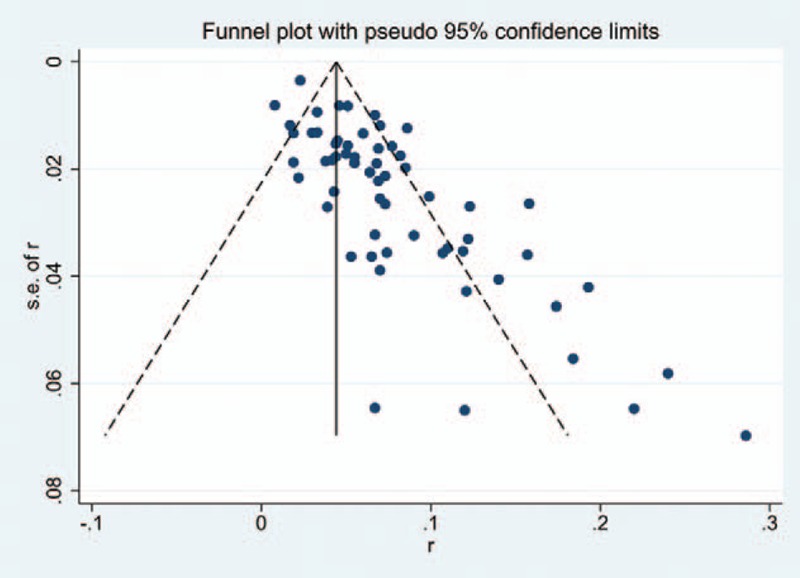
Funnel plot showing incidence of C5 for after cervical surgery. CI = confidence interval, df = degrees of freedom, M–H = Mantel–Haenszel.

According to the shape of the funnel plot (Fig. 22) and *P* value (*P* = .557,.117) of the Begg and Egger regression tests, no visually asymmetrical and statistical evidence of publication bias of included studies is revealed. Likewise, for subgroup analysis, publication bias was also not found in included studies.

## 4 Discussion

A number of studies focused on the occurrence of C5 palsy after cervical surgery. Even though some mechanisms trying to explain this common complication have been proposed, it remains a controversial issue. C5 palsy after cervical surgery is considered to be a result of nerve root injury or segmental spinal cord disorder.^[64]^ As some conditions may contribute to C5 palsy, we reviewed 5 pathologic mechanisms as follows: inadvertent injury to the nerve root during surgery;^[79]^ shifting of the cord caused nerve root traction after surgery;^[80]^ spinal cord ischemia caused by decreased blood supply;^[81]^ segmental spinal cord disorder;^[82]^ and reperfusion injury of the spinal cord.^[83]^

Several meta-analyses reported on the incidence of C5 palsy. Shou et al^[2]^ performed a meta-analysis focused on the epidemiological prevalence estimates of C5 palsy following cervical surgery and it was based on 13,621 patients from 79 articles. He concluded that cervical surgery is associated with C5 palsy, particularly in patients who received LF and male patients are risk factors of C5 palsy. Gu^[16]^ explored the incidence and risk factors of C5 palsy after posterior cervical surgery by a meta-analysis and found that patients with excessive spinal cord drift, preexisting intervertebral foramenal stenosis, OPLL, laminectomy, and male patients were risk factors for C5 palsy. Gu just assessed the C5 palsy after posterior approaches. We conducted a meta-analysis on C5 palsy for the last decade.

The results showed that 721 with C5 palsy from total 11,481 patients (6.3%) after cervical surgery in 61 included articles. Posterior approaches have a higher incidence of C5 palsy than that in anterior approached. Among all the procedures, occurrences of C5 palsy after ACDF, ACCF, ACCDF, LP, LF were 5.5%, 7.5%, 6%, 4.4%, and 12.2%. In ACDF and LP, patients with OPLL (5.5%, 8.1%, respectively) have a higher incidence than in patients with CSM (4.7%, 3.1%, respectively); however, in LF, patients with CSM and with OPLL have similar incidence of C5 palsy (13% vs 13.1%). In most cases, C5 palsy was unilateral (74.5%).

### 4.1 Anterior approaches versus posterior approaches

Bydon et al^[84]^ reported on comparison between anterior and posterior approaches, the incidence of C5 palsy was significantly higher in the posterior method than the anterior method (8.6% vs 1.6%) (*P*<.001). Chang et al^[40]^ performed another study to compare anterior and posterior approaches. In their study, the incidence of C5 palsy has been reported as 0.7% and 8.8% for anterior and posterior, respectively. Chen et al^[24]^ reported the incidence of C5 palsy following posterior surgical routes as 24.3% and 0% following anterior surgical routes. In the same study, the highest incidence of C5 palsy of all published studies has been reported (25% for posterior laminectomy and fusion). These studies revealed that anterior surgical routes for patients had lower risk of developing postoperative C5 palsy. Shou et al^[2]^ suggested that patients who received posterior cervical surgery (5.8%) had a slightly higher prevalence than patients who underwent anterior surgery (5.2%). Our results showed that posterior approaches (6.2%) had a higher incidence of C5 palsy, compared with anterior approaches (5%). We had a similar trend with Shou's, but we had difference of the incidence of C5 palsy after anterior and posterior approaches, this may be relation with difference of year for included studies. Our included studies were derived from last decade, but Shou's were from 1989 to 2014, which might lead to the difference. As we know, posterior shift of the spinal cord at C4–5 in posterior group has been significantly greater than that in anterior group. Nakashima et al^[54]^ reported that C5 palsy was caused by posterior shift of the spinal cord and additional iatrogenic foraminal stenosis due to cervical alignment correction after posterior instrumentation with fusion.

### 4.2 Different anterior procedures

Liu et al^[50]^ accessed complications of different techniques anterior decompression and found that patients who underwent multilevel corpectomy group had the highest incidence of C5 palsy (11.9%). Lin et al^[49]^ explored the same topic on comparison of ACDF and ACCF in patients with multilevel CSM. Their conclusions revealed that the incidence of C5 palsy was 3.5% in ACDF and 4.8% in ACCF. ACCF had higher incidence of C5 palsy. In our meta-analysis, patients who received ACDF had the lowest incidence of C5 palsy (5.5%) and ACCF was reported as the highest prevalence (7.5%) in anterior group. ACDF could preserve more vertebral body and provide more points of distraction and fixation except for the graft and interbody space shaping than these of ACCF. ACCF may lead to significant drift of spinal cord away from ventral side.

### 4.3 Different posterior procedures

Posterior cervical decompression, LP and LF, is a well-recognized surgical approach for multilevel CSM or OPLL. Yang et al^[19]^ believed that the C5 palsy rate in the LP group is significantly higher than that in the LF group. However, Xia et al^[85]^ had diverse point that there was no significant difference between LP and LF. Although many studies reported on the occurrence of C5 palsy after posterior cervical decompression, its detailed mechanism remained poorly understood. Our results showed that the incidence of C5 palsy was significant higher in LF, which was similar to results of previous meta-analysis, and suggested LF as a significant risk factor. That may be because the LF removes the intact posterior arch of the vertebra, thus providing an excessive space for the spinal cord to shift posteriorly and showing greater change in dural sac area. For this reason, we considered LP as a more viable posterior option for patients with CSM or OPLL.

### 4.4 CSM and OPLL

We evaluated incidence of C5 palsy for patients with CSM and OPLL. We found that patients with CSM (4.8%) had a lower incidence of C5 palsy than patients with OPLL (8.1%). We also assessed the incidence of C5 palsy for CSM or OPLL in ACDF, LP, or LF. The consequences showed that incidences of C5 palsy for CSM in ACDF, LP, and LF were 4.7%, 3.1%, 13%, respectively and for OPLL were 5.5%, 8.1%, 13.1%, respectively. The incidence of C5 palsy for CSM group by ACDF and LP was lower than these in the OPLL group; nevertheless, the prevalence for both CSM group and OPLL group in LF was similar. Above all suggested that patients with OPLL, compared with patients with CSM, were more susceptible to this complication.

### 4.5 Sex and sides for C5 palsy

Our results revealed that male patients (5.9%), compared with female patients (4.1%), were more likely to have C5 palsy, which were similar to Shou's. In most cases, C5 palsy was unilateral.

### 4.6 Limitations

There were several limitations of this study. First, there was no RCT on C5 palsy, we need RCT to further study; Second, the statistical power could be improved in the future by including more studies. Some parameters, like 1-level CSM for C5 palsy, 2-level CSM for C5 palsy, or multilevel CSM for C5 palsy, due to the lack of data could not be analyzed by subgroups to avoid a high heterogeneity which may exert instability on the consistency of the outcomes; Third, the searching strategy was restricted to articles published in the English and Chinese languages. Articles with potentially high-quality data that were published in other languages were not included because of anticipated difficulties in obtaining accurate medical translations.

In summary, in posterior approaches, male patients and patients with OPLL have a higher incidence of C5 palsy. In ACDF and LP, patients with OPLL had a higher incidence of C5 palsy, but in LF, both patients with CSM and OPLL had similar incidence of C5 palsy. Future more studies with high methodological quality are needed to evaluate incidence of C5 palsy.

## References

[1] Scoville WB Cervical spondylosis treated by bilateral facetectomy and laminectomy. J Neurosurg 1961;18:423–8. 13749416 10.3171/jns.1961.18.4.0423

[2] Shou F Li Z Wang H Prevalence of C5 nerve root palsy after cervical decompressive surgery: a meta-analysis. Eur Spine J 2015;24:2724–34. 26281981 10.1007/s00586-015-4186-5

[3] Sakaura H Hosono N Mukai Y C5 palsy after decompression surgery for cervical myelopathy. Spine (Phila Pa 1976) 2006;28:2447–51. 10.1097/01.BRS.0000090833.96168.3F 14595162

[4] Anderson PA Matz PG Groff MW Laminectomy and fusion for the treatment of cervical degenerative myelopathy. Spine (Phila Pa 1976) 2009;11:150–6. 10.3171/2009.2.SPINE08727 19769494

[5] Tanaka N Nakanishi K Fujiwara Y Postoperative segmental C5 palsy after cervical laminoplasty may occur without intraoperative nerve injury: a prospective study with transcranial electric motor-evoked potentials. Spine (Phila Pa 1976) 2006;31:3013–7. 17172998 10.1097/01.brs.0000250303.17840.96

[6] Luo J Cao K Huang S Comparison of anterior approach versus posterior approach for the treatment of multilevel cervical spondyloticmyelopathy. Eur Spine J 2015;24:1621–30. 25840781 10.1007/s00586-015-3911-4

[7] Guzman JZ Baird EO Fields AC C5 nerve root palsy following decompression of the cervical spine: a systematic evaluation of the literature. Bone Joint J 2014;96-B:950–5. 24986950 10.1302/0301-620X.96B7.33665

[8] Gu YF Cao P Gao R Incidence and risk factors of C5 palsy following posterior cervical decompression: a systematic review. PLoS One 2014;9:e101933. 25162509 10.1371/journal.pone.0101933 PMC4146468

[9] Tsuzuki N Abe R Saiki K Paralysis of the arm after posterior decompression of the cervical spinal cord. Analysis of clinical findings. Eur Spine J 1993;2:197–202. 20058405 10.1007/BF00299446

[10] Sasai K Saito T Akagi S Preventing C5 palsy after laminoplasty. Spine (Phila Pa 1976) 2003;28:1972–7. 12973145 10.1097/01.BRS.0000083237.94535.46

[11] Uematsu Y Tokuhashi Y Matsuzaki H Radiculopathy after laminoplasty of the cervical spine. Spine (Phila Pa 1976) 1998;23:2057–62. 9794049 10.1097/00007632-199810010-00004

[12] Komagata M Nishiyama M Endo K Prophylaxis of C5 palsy after cervical expansive laminoplasty by bilateral partial foraminotomy. Spine J 2004;4:650–5. 15541697 10.1016/j.spinee.2004.03.022

[13] Hasegawa K Homma T Chiba Y Upper extremity palsy following cervical decompression surgery results from a transient spinal cord lesion. Spine (Phila Pa 1976) 2007;32:E197–202. 17413460 10.1097/01.brs.0000257576.84646.49

[14] Matsunaga H Inada M Takeuchi M Pathogenesis and prevention of C5 palsy after cervical laminoplasty [in Japanese]. Chubu Jpn Orthop Trauma Surg 2007;50:135–6.

[15] Basaran R Kaner T C5 nerve root palsy following decompression of cervical spine with anterior versus posterior types of procedures in patients with cervical myelopathy. Eur Spine J 2016;25:2050–9. 27095700 10.1007/s00586-016-4567-4

[16] Higgins JP Thompson SG Deeks JJ Measuring inconsistency in meta-analyses. BMJ 2003;327:557–60. 12958120 10.1136/bmj.327.7414.557 PMC192859

[17] Mantel N Haenszel W Statistical aspects of the analysis of data from retrospective studies of disease. J Natl Cancer Inst 1959;22:719–48. 13655060

[18] Chen Y Liu X Chen D Surgical strategy for ossification of the posterior longitudinal ligament in the cervical spine. Orthopedics 2012;35:e1231–7. 22868611 10.3928/01477447-20120725-25

[19] Yang L Gu Y Shi J Modified plate-only open-door laminoplasty versus laminectomy and fusion for the treatment of cervical stenotic myelopathy. Orthopedics 2013;36:e79–E87. 23276358 10.3928/01477447-20121217-23

[20] Kaneyama S Sumi M Kanatani T Prospective study and multivariate analysis of the incidence of C5 palsy after cervical laminoplasty. Spine (Phila Pa 1976) 2010;35:E1553–8. 21116219 10.1097/BRS.0b013e3181ce873d

[21] Katsumi K Yamazaki A Watanabe K Can prophylactic bilateral C4/C5 foraminotomy prevent postoperative C5 palsy after open-door laminoplasty?: a prospective study. Spine (Phila Pa 1976) 2012;37:748–54. 21912316 10.1097/BRS.0b013e3182326957

[22] Odate S Shikata J Yamamura S Extremely wide and asymmetric anterior decompression causes postoperative C5 palsy: an analysis of 32 patients with postoperative C5 palsy after anterior cervical decompression and fusion. Spine (Phila Pa 1976) 2013;38:2184–9. 24108301 10.1097/BRS.0000000000000019

[23] Lee SH Suk KS Kang KC Outcomes and related factors of C5 palsy following cervical laminectomy with instrumented fusion compared with laminoplasty. Spine (Phila Pa 1976) 2016;41:E574–9. 26650877 10.1097/BRS.0000000000001343

[24] Chen Y Guo Y Lu X Surgical strategy for multilevel severe ossification of posterior longitudinal ligament in the cervical spine. J Spinal Disord Tech 2011;24:24–30. 20924295 10.1097/BSD.0b013e3181c7e91e

[25] Takemitsu M Cheung KM Wong YW C5 nerve root palsy after cervical laminoplasty and posterior fusion with instrumentation. J Spinal Disord Tech 2008;21:267–72. 18525487 10.1097/BSD.0b013e31812f6f54

[26] Katsumi K Yamazaki A Watanabe K Analysis of C5 palsy after cervical open-door laminoplasty: relationship between C5 palsy and foraminal stenosis. J Spinal Disord Tech 2013;26:177–82. 22124424 10.1097/BSD.0b013e31823db346

[27] Chen Y Wang X Chen D Posterior hybrid technique for ossification of the posterior longitudinal ligament associated with segmental instability in the cervical spine. J Spinal Disord Tech 2014;27:240–4. 22576720 10.1097/BSD.0b013e31825c6e2f

[28] Park JH Roh SW Rhim SC Long-term outcomes of 2 cervical laminoplasty methods: midline splitting versus unilateral single door. J Spinal Disord Tech 2012;25:E224–9. 23160272 10.1097/BSD.0b013e31825dda6b

[29] Kim S Lee SH Kim ES Clinical and radiographic analysis of c5 palsy after anterior cervical decompression and fusion for cervical degenerative disease. J Spinal Disord Tech 2014;27:436–41. 22832559 10.1097/BSD.0b013e31826a10b0

[30] Odate S Shikata J Kimura H Hybrid decompression and fixation technique versus plated three-vertebra corpectomy for four-segment cervical myelopathy: analysis of 81 cases with a minimum 2-year follow-up. J Spinal Disord Tech 2012;8:36–41. 10.1097/BSD.0b013e31827ada34 23168392

[31] Nakamae T Tanaka N Nakanishi K Investigation of segmental motor paralysis after cervical laminoplasty using intraoperative spinal cordmonitoring with transcranial electric motor-evoked potentials. J Spinal Disord Tech 2012;25:92–8. 22454184 10.1097/BSD.0b013e318211fc4e

[32] Zhang H Lu S Sun T Effect of lamina open angles in expansion open-door laminoplasty on the clinical results in treating cervical spondylotic myelopathy. J Spinal Disord Tech 2015;28:89–94. 22832551 10.1097/BSD.0b013e3182695295

[33] Cherian J Mayer RR Haroun KB Contribution of Lordotic correction on C5 palsy following cervical laminectomy and fusion. Neurosurgery 2016;79:816–22. 26813859 10.1227/NEU.0000000000001199

[34] Meng H Fang X Hao D Incidences of C5 nerve palsy after multi-segmental cervical decompression through different approaches. Nan Fang Yi Ke Da Xue Xue Bao 2015;35:315–8. 25818772

[35] Liu X Wang H Zhou Z Anterior decompression and fusion versus posterior laminoplasty for multilevel cervical compressive myelopathy. Orthopedics 2014;37:e117–22. 24679196 10.3928/01477447-20140124-12

[36] Bai CR Wang BQ Li KH Benefit of degenerative posterior longitudinal ligament removal during anterior decompression in cervical spondylotic myelopathy. Orthopedics 2015;38:e54–61. 25611421 10.3928/01477447-20150105-61

[37] Blizzard DJ Gallizzi MA Sheets C The role of iatrogenic foraminal stenosis from lordotic correction in the development of C5 palsy after posterior laminectomy and fusion. J Orthop Surg Res 2015;10:160. 26438515 10.1186/s13018-015-0297-2 PMC4595268

[38] Bydon M Macki M Aygun N Development of postoperative C5 palsy is associated with wider posterior decompressions: an analysis of 41patients. Spine J 2014;14:2861–7. 24704500 10.1016/j.spinee.2014.03.040

[39] Campbell PG Yadla S Malone J Early complications related to approach in cervical spine surgery: single-center prospective study. World Neurosurg 2010;74:363–8. 21492571 10.1016/j.wneu.2010.05.034

[40] Chang PY Chan RC Tsai YA Quantitative measures of functional outcomes and quality of life in patients with C5 palsy. J Chin Med Assoc 2013;76:378–84. 23664730 10.1016/j.jcma.2013.03.008

[41] Eskander MS Balsis SM Balinger C The association between preoperative spinal cord rotation and postoperative C5 nerve palsy. J Bone Joint Surg Am 2012;94:1605–9. 22992851 10.2106/JBJS.K.00664

[42] Guo Q Ni B Zhou F Anterior hybrid decompression and segmental fixation for adjacent three-level cervical spondylosis. Arch Orthop Trauma Surg 2011;131:631–6. 20809065 10.1007/s00402-010-1181-5

[43] Hashimoto M Mochizuki M Aiba A C5 palsy following anterior decompression and spinal fusion for cervical degenerative diseases. Eur Spine J 2010;19:1702–10. 20461418 10.1007/s00586-010-1427-5 PMC2989233

[44] Wang H Zhang X Lv B Analysis of correlative risk factors for C5 palsy after anterior cervical decompression and fusion. Int J Clin Exp Med 2015;8:3983–91. 26064300 PMC4443134

[45] Imagama S Matsuyama Y Yukawa Y C5 palsy after cervical laminoplasty: a multicentre study. J Bone Joint Surg Br 2010;92:393–400. 20190311 10.1302/0301-620X.92B3.22786

[46] Kanchiku T Imajo Y Suzuki H Results of surgical treatment of cervical spondylotic myelopathy in patients aged 75 years or more: a comparative study of operative methods. Arch Orthop Trauma Surg 2014;134:1045–50. 24880218 10.1007/s00402-014-2017-5

[47] Kurakawa T Miyamoto H Kaneyama S C5 nerve palsy after posterior reconstruction surgery: predictive risk factors of the incidence and critical range of correction for kyphosis. Eur Spine J 2016;25:2060–7. 27055443 10.1007/s00586-016-4548-7

[48] Lim CH Roh SW Rhim SC Clinical analysis of C5 palsy after cervical decompression surgery: relationship between recovery duration and clinical and radiological factors. Eur Spine J 2016;21:474–81. 10.1007/s00586-016-4664-4 27342613

[49] Lin Q Zhou X Wang X A comparison of anterior cervical discectomy and corpectomy in patients with multilevel cervical spondylotic myelopathy. Eur Spine J 2012;21:474–81. 21826497 10.1007/s00586-011-1961-9 PMC3296841

[50] Liu Y Qi M Chen H Comparative analysis of complications of different reconstructive techniques following anterior decompression for multilevel cervical spondylotic myelopathy. Eur Spine J 2012;21:2428–35. 22644433 10.1007/s00586-012-2323-y PMC3508223

[51] Lubelski D Derakhshan A Nowacki AS Predicting C5 palsy via the use of preoperative anatomic measurements. Spine J 2014;14:1895–901. 24225009 10.1016/j.spinee.2013.10.038

[52] Miller JA Lubelski D Alvin MD C5 palsy after posterior cervical decompression and fusion: cost and quality-of-life implications. Spine J 2014;14:2854–60. 24704502 10.1016/j.spinee.2014.03.038

[53] Minamide A Yoshida M Yamada H Clinical outcomes of microendoscopic decompression surgery for cervical myelopathy. Eur Spine J 2010;19:487–93. 19956984 10.1007/s00586-009-1233-0 PMC2899765

[54] Nakashima H Imagama S Yukawa Y Multivariate analysis of C-5 palsy incidence after cervical posterior fusion with instrumentation. J Neurosurg Spine 2012;17:103–10. 22632173 10.3171/2012.4.SPINE11255

[55] Chen Y Chen D Wang X C5 palsy after laminectomy and posterior cervical fixation for ossification of posterior longitudinal ligament. J Spinal Disord Tech 2007;20:533–5. 17912131 10.1097/BSD.0b013e318042b655

[56] Sun T-w Zhang H Lu S-l Clinical analysis of C5 nerve root palsy in hinge side and different andles in lamina open-door after expansion of open-door cervical laminoplasty. Chin J Reparative Reconstr Surg 2011;11:1285–9. 22229177

[57] Nassr A Eck JC Ponnappan RK The incidence of C5 palsy after multilevel cervical decompression procedures: a review of 750 consecutive cases. Spine (Phila Pa 1976) 2012;37:174–8. 22293780 10.1097/BRS.0b013e318219cfe9

[58] Chugh AJ Gebhart JJ Eubanks JD Predicting postoperative C5 palsy using preoperative spinal cord rotation. Orthopedics 2015;38:e830–5. 26375543 10.3928/01477447-20150902-63

[59] Macki M Alam R Kerezoudis P Manual muscle test at C5 palsy onset predicts the likelihood of and time to C5 palsy resolution. J Clin Neurosci 2016;24:112–6. 26602799 10.1016/j.jocn.2015.09.003

[60] Kotil K Tari R Two level cervical corpectomy with iliac crest fusion and rigid plate fixation: a retrospective study with a three-year follow-up. Turk Neurosurg 2011;21:606–12. 22194123

[61] Wu FL Sun Y Pan SF Risk factors associated with upper extremity palsy after expansive open-door laminoplasty for cervical myelopathy. Spine J 2014;14:909–15. 24120145 10.1016/j.spinee.2013.07.445

[62] Yamanaka K Tachibana T Moriyama T C-5 palsy after cervical laminoplasty with instrumented posterior fusion. J Neurosurg Spine 2014;20:1–4. 24160298 10.3171/2013.9.SPINE12952

[63] Wang M-y Zhang S-y Risk factors associated with C5 palsy after cervical posterior single door vertebral canal plasty. Shanxi Medical University.

[64] Zhang B Dai M Tang Y Surgical treatment of ossification of the posterior longitudinal ligament of cervical spine. Orthop J China 2011;19:1601–4.

[65] Wang L Wang G-h Clinical observation of laminectomy and fusion for cervical spondylotic myelopathy. Hainan Med J 2014;25:1498–9.

[66] Duan G Jia X-h Ran B Effect of cervical curvature change on C5 nerve root palsy after anterior cervical decompression and fusion. Orthoped J China 2015;23:1169–72.

[67] Li Q-y Hu J-h Tian Y Clinical observation and Analysis of C5 palsy after cervical surgery. Chin J Bone Joint Surg 2012;5:433–6.

[68] Jia B Zhou X-q Zhang C-j The comparison of C5 palsy after two posterior cervical operations on multilevel cervical spondylotic myelopathy. China Med Herald 2016;13:85–8.

[69] Wu W Zhu T-L Comparison of anterior cervical discectomy and fusion and anterior cervical corpectomy and fusion for the treatment of multi-segmental cervical spondylotic myelopathy. J Clin Orthop 2014;17:497–500.

[70] Wang X Wei M-k Liang B Comparison of clinic outcome of severe cervical ossification of the posterior longitudinal ligament (OPLL) between laminoplasty and posterior laminoplasty (or laminotomy) combined with anterior decompression and fusion. Orthoped J China 2013;21:2138–41.

[71] Liu Y Hou Y Yang L Comparison of 3 reconstructive techniques in the surgical management of multilevel cervical spondylotic myelopathy. Spine (Phila Pa 1976) 2012;37:E1450–8. 22869063 10.1097/BRS.0b013e31826c72b4

[72] Guo Q Bi X Ni B Outcomes of three anterior decompression and fusion techniques in the treatment of three-level cervical spondylosis. Eur Spine J 2011;20:1539–44. 21448583 10.1007/s00586-011-1735-4 PMC3175896

[73] Lian XF Xu JG Zeng BF Noncontiguous anterior decompression and fusion for multilevel cervical spondylotic myelopathy: a prospective randomized control clinical study. Eur Spine J 2010;19:713–9. 20174838 10.1007/s00586-010-1319-8 PMC2899955

[74] Liu J Chen X Liu Z Anterior cervical discectomy and fusion versus corpectomy and fusion in treating two-level adjacent cervical spondylotic myelopathy: a minimum 5-year follow-up study. Arch Orthop Trauma Surg 2015;135:149–53. 25424752 10.1007/s00402-014-2123-4

[75] Qi M Wang X-w Liu Y Comparative analysis of complications of different anterior decompression procedures for treating multilevel cervical spondylotic myelopathy. Chin J Spine Spinal Cord 2012;22:963–8. 10.1007/s00586-012-2323-y PMC3508223 22644433

[76] Chen G Luo Z Nalajala B Expansive open-door laminoplasty with titanium miniplate versus sutures. Orthopedics 2012;35:e543–8. 22495857 10.3928/01477447-20120327-24

[77] Li Z Huang J Zhang Z A comparison of multilevel anterior cervical discectomy and corpectomy in patients with 4-level cervical spondylotic myelopathy: a minimum 2-year follow-up study. Clin Spine Surg 2016;[Epub ahead of print]. 10.1097/BSD.0000000000000212 28525475

[78] Hu Y Zhao H-y Dong W-x Comparative study of laminoplasty and laminectomy combined fusion for treatment of multi-level cervical myelopathy. J Spinal Surg 2014;12:226–30.

[79] Tsuzuki N Abe R Saiki K Extradural tethering effect as one mechanism of radiculopathy complicating posterior decompression of the cervical spinal cord. Spine (Phila Pa 1976) 1996;21:203–10. 8720405 10.1097/00007632-199601150-00008

[80] Sakaura H Hosono N Mukai Y C5 palsy after decompression surgery for cervical myelopathy: review of the literature. Spine 2003;28:2447–51. 14595162 10.1097/01.BRS.0000090833.96168.3F

[81] Hirabayashi K Satomi K Operative procedure and results of expansive open-door laminoplasty. Spine (Phila Pa 1976) 1988;13:870–6. 3143157 10.1097/00007632-198807000-00032

[82] Komagata M Nishiyama M Endoh K Clinical study of the post operative C5 palsy after cervical laminoplasty; efficacy of bilateral partial foraminotomy for prevention the C5 palsy. J Jpn Spine Res Soc 2002;131:237.

[83] Shimizu T Shimada H Edakuni H Post-laminoplasty palsy of upper extremities, with special reference to the spinal cord factors. Bessatsu Seikeigeka 1996;29:188–93.

[84] Bydon M Macki M Kaloostian P Incidence and prognostic factors of c5 palsy: a clinical study of 1001 cases and review of the literature. Neurosurgery 2014;74:595–604. 24561867 10.1227/NEU.0000000000000322

[85] Xia G Tian R Xu T Spinal posterior movement after posterior cervical decompression surgery: clinical findings and factors affecting postoperative functional recovery. Orthopedics 2011;34:e911–8. 22146210 10.3928/01477447-20111021-03

